# Irritative ventricular tachycardia after transcatheter pulmonary valve replacement

**DOI:** 10.1093/ehjcr/ytag029

**Published:** 2026-02-03

**Authors:** Victor Waldmann, Antoine Legendre, Pauline Pinon, Sophie-Guiti Malekzadeh-Milani, Damien Bonnet

**Affiliations:** Université Paris Cité, PARCC, INSERM U970, 56 rue Leblanc, Paris 75015, France; Division of Cardiology, European Georges Pompidou Hospital, 20-40 Rue Leblanc, 75908 Paris Cedex 15, Paris, France; M3C-Cardiologie Pédiatrique, F-AP-HP, Groupe Hospitalier Universitaire Centre, Hôpital Universitaire Necker Enfants Malades, Université Paris Cité, 149 rue de Sèvres, 75015 Paris, France; Division of Cardiology, European Georges Pompidou Hospital, 20-40 Rue Leblanc, 75908 Paris Cedex 15, Paris, France; M3C-Cardiologie Pédiatrique, F-AP-HP, Groupe Hospitalier Universitaire Centre, Hôpital Universitaire Necker Enfants Malades, Université Paris Cité, 149 rue de Sèvres, 75015 Paris, France; Division of Cardiology, European Georges Pompidou Hospital, 20-40 Rue Leblanc, 75908 Paris Cedex 15, Paris, France; M3C-Cardiologie Pédiatrique, F-AP-HP, Groupe Hospitalier Universitaire Centre, Hôpital Universitaire Necker Enfants Malades, Université Paris Cité, 149 rue de Sèvres, 75015 Paris, France; M3C-Cardiologie Pédiatrique, F-AP-HP, Groupe Hospitalier Universitaire Centre, Hôpital Universitaire Necker Enfants Malades, Université Paris Cité, 149 rue de Sèvres, 75015 Paris, France; M3C-Cardiologie Pédiatrique, F-AP-HP, Groupe Hospitalier Universitaire Centre, Hôpital Universitaire Necker Enfants Malades, Université Paris Cité, 149 rue de Sèvres, 75015 Paris, France

A 17-year-old patient with repaired tetralogy of Fallot underwent transcatheter pulmonary valve (TPV) replacement in December 2024 using a self-expanding Venus P-Valve (Venus Medtech, Hangzhou, China) for severe pulmonary regurgitation associated with significant right ventricular dilatation. Prior to the procedure, no ventricular arrhythmia was documented, and the patient was not receiving any antiarrhythmic medication. In April 2025, an electrophysiological study (EPS) was performed following an episode of exertional syncope to investigate a potential arrhythmic cause. A sustained ventricular tachycardia (VT) was reproducibly inducible. Haemodynamic tolerance was acceptable, allowing rapid high-density activation 3D mapping, which identified a fast (300 b.p.m.) focal VT originating from the proximal part of the TPV (*Panels A* and *B*; Supplementary material online, *Video S1*). Multiple and prolongated irrigated radiofrequency applications were required before tachycardia was no longer inducible. As the patient declined implantable cardioverter-defibrillator implantation, he was discharged with a LifeVest wearable cardioverter-defibrillator. Although the patient remained asymptomatic, a repeat EPS was performed 3 months later to confirm durable non-inducibility. The same VT was however inducible with aggressive ventricular stimulation, and additional radiofrequency applications were performed. At the end of this second procedure, the VT was no longer inducible, and the patient has remained free of clinical arrhythmia during 6 months of follow-up (under nadolol therapy). An implantable loop recorder was implanted for close monitoring of any potential arrhythmic event, and no ventricular arrhythmia was detected. Although current data are limited, the potential risk of ventricular arrhythmias after TPV implantation is increasingly recognized.^1–3^ These are thought to result from contraction–excitation feedback induced by myocardial stretch after stent deployment. New generation TPV devices are designed to accommodate native, patched, and enlarged right ventricular outflow tract, and some degree of stent protrusion into the subpulmonary outflow tract can occur, especially in patients with a short pulmonary trunk (as observed in this patient). This case perfectly illustrates the very likely irritative mechanism of the VT and also highlights the potential complexity of ablating the underlying substrate located at or just beneath the proximal part of the valve. Considering the important and continuous growing use of TPV over surgical valve replacement across various anatomies, further studies are essential to better assess arrhythmic outcomes among the different TPVs and between TPV and surgical replacement and to establish best strategies for prevention, monitoring, and treatment of related arrhythmias.

**Figure:**
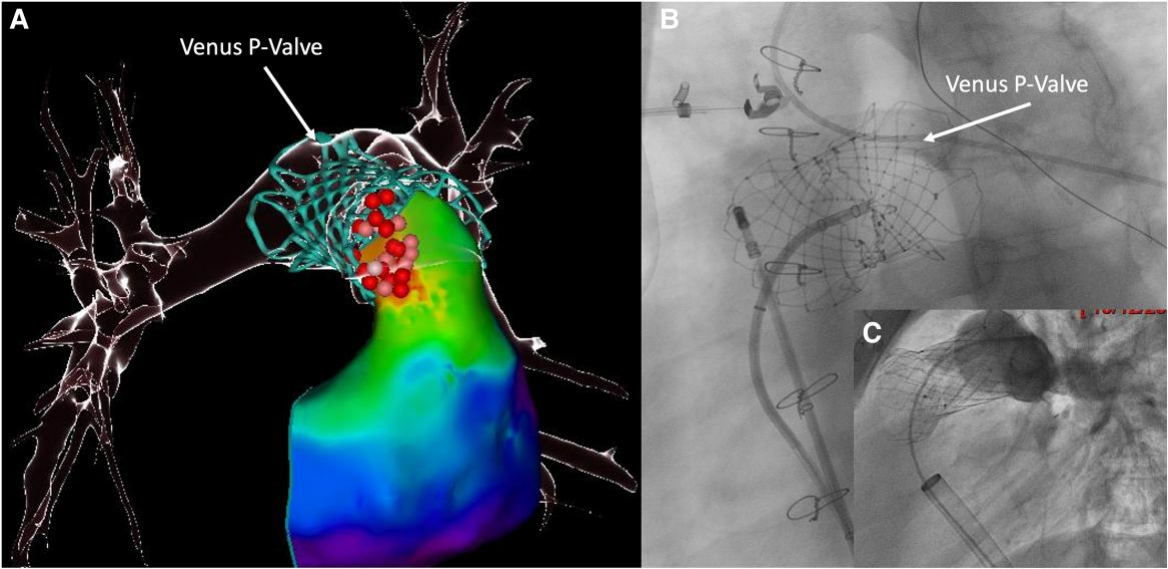


## Supplementary Material

ytag029_Supplementary_Data

## Data Availability

The data underlying this article are available in the article.
